# crm12comb: Phase I/II adaptive design for drug combinations based on CRM design through R

**DOI:** 10.1371/journal.pone.0336146

**Published:** 2025-11-10

**Authors:** Junying Wang, Song Wu, Jie Yang

**Affiliations:** 1 Department of Applied Mathematics and Statistics, Stony Brook University, Stony Brook, United States of America; 2 Department of Family, Population and Preventive Medicine, Stony Brook University, Stony Brook, United States of America; UTRGV: The University of Texas Rio Grande Valley, UNITED STATES OF AMERICA

## Abstract

Adaptive designs for integrated phase I/II trials of drug combinations are increasingly utilized to speed up the drug development process and enhance drug efficacy, particularly in the realm of cancer therapy. The model-based Continual Reassessment Method (CRM) for dose-finding is widely used to leverage accumulated data in guiding patient allocation, drawing on principles from the Bayesian framework. In this paper, we present **crm12Comb**, an R package we developed to streamline the Phase I/II adaptive design process for drug combinations using the CRM approach. This package supports patients’ assignment guidance in a single trial based on existing data, as well as simulation studies for conducting extensive simulations with multiple trial parameters to evaluate operating characteristics and create visual representations. It accounts for toxicity and efficacy as binary outcomes, applying partial orderings to the dose-toxicity and dose-efficacy relationships for drug combinations. **crm12Comb** allows for a wide range of user-specified parameters, including maximum number of patients, cohort size, drug combinations, and a variety of link functions with prior distributions, offering flexibility to accommodate diverse clinical scenarios.

## Introduction

In general, phase I dose-finding designs aim to find the maximum tolerated dose (MTD), which will be used as the recommended dose in the following phase II trials to assess the corresponding efficacy. The MTD is defined as the highest dose level with acceptable toxicity. In most oncology trials, the determination of the MTD involves considering dose-limiting toxicity (DLT). Traditionally, phase I and phase II trials are conducted in a separate and sequential manner. However, the recent trend has seen a rise in the integration of phase I and II trials, primarily due to the significant benefits this approach offers. These benefits include expediting the drug development timeline, minimizing the required number of participants, and ethically optimizing patient allocation to more efficacious dosages. Furthermore, the use of drug combinations in cancer therapy is a critical strategy to avert drug resistance and effectively target cancer cells. To this end, numerous clinical trial designs have been put forward to maximize these advantages.

Traditionally, phase I dose-finding designs have relied on algorithmic approaches, such as 3 + 3 design [1] and accelerated titration design [2], which utilize pre-specified rules to guide dose escalation/de-escalation. Despite their relatively modest performance, these algorithm-based designs are popular in practice [3] due to their simplicity and low DLT rates. To enhance operating effectiveness, model-based designs have been developed. These designs, such as continual reassessment method (CRM) [4] and escalation with overdose control (EWOC) [5], use accumulated data to estimate the dose-toxicity curve, offering more flexible and more accurate dose allocation for each patient cohort by utilizing all available information. However, these phase I dose-finding designs typically focus only on toxicity without considering efficacy and are in the context of single-drug escalations.

Meanwhile, various methods have been developed for dose-finding designs in drug combination trials. For example, linear programming has been employed to identify the optimal combined dose within the boundary of acceptable toxicity [6]. A plethora of Bayesian-based designs have been extensively applied to drug combinations [7–13]. Despite the diversity of these designs, a common limitation is their lack of consideration for efficacy alongside toxicity.

To assess both toxicity and efficacy simultaneously, the integrated phase I/II trials have been proposed, incorporating various definitions of toxicity and efficacy outcomes, such as methods that are based on ordinal outcome [14] and the correlated bivariate binary outcome [15] for single drugs. To accommodate for drug combinations, recent advancements have led to the proposal of various designs specifically tailored to incorporate drug combinations in integrated phase I/II trials, as demonstrated by recent works [16–20]. One practical approach to addressing drug combinations were to introduce the concept of an acceptable set [21,22]. In this approach, a fixed acceptable set is constructed based on phase I data to exclude those overly toxic doses in the phase II trial when assessing efficacy. Wages and Conaway [23] modified this concept to allow for continuous updates of acceptable drug combinations, aligning the efficacy assessment with the CRM design. This dynamic approach has been successfully implemented in real clinical trials [24,25]. However, their design assumed only the empiric link function with a standard normal prior distribution for estimating the toxicity and efficacy. In real-world scenarios, various combinations of link functions and prior distributions could be considered.

The CRM designs have gained widespread applications, bolstered by the development of various software tools that leverage their strong performances. The intricate nature of CRM methodologies underscores the substantial demand for software solutions that offer clinicians insights and simulations across different CRM design configurations. In the R programming environment, several packages have been developed to facilitate Phase I CRM designs, including CRM [26] which provides MTD and operating characteristics from simulations, dfcrm [27], which employs Bayesian inference without using Markov chain Monte Carlo (MCMC) sampling, and crmPack [28] and bcrm [29], which utilize MCMC sampling via JAGS/OpenBUGS/WinBUGS. Additionally, trialr [30] employs MCMC sampling via Stan [31,32], and pocrm [33] implements partial orderings for drug combinations. While these packages have been widely used, CRM/dfcrm/crmPack/bcrm can perform CRM design focusing only on toxicity with single drugs, pocrm utilizes partial ordering to drug combinations but only designed via toxicity, and trialr further includes efficacy but lacks considering the drug combinations. Currently, no R package can comprehensively cover both toxicity and efficacy for drug combinations.

In this paper, we address the existing gap by delving into a CRM-based phase I/II adaptive design for drug combinations that incorporate both toxicity and efficacy and include a wide range of link functions and prior distributions. We present an R package named crm12Comb, designed to facilitate the whole process and is publicly available via the Comprehensive R Archive Network (CRAN) at https://cran.r-project.org/web/packages/crm12Comb/. The crm12Comb package allows for simulation studies to be conducted before the trial commences, providing a general performance overview, or for estimating the next drug combination allocation based on current data.

## Method

The initial methodology was proposed by Wages and Conaway [23], which considered the empiric link function with a standard normal prior distribution for the phase I/II adaptive design for drug combinations using CRM design. This approach involves constructing an acceptable set based on the estimated toxicity probabilities and determining the allocation of the next patient or patient cohort based on the estimated efficacy probabilities.

**Fig 1:**
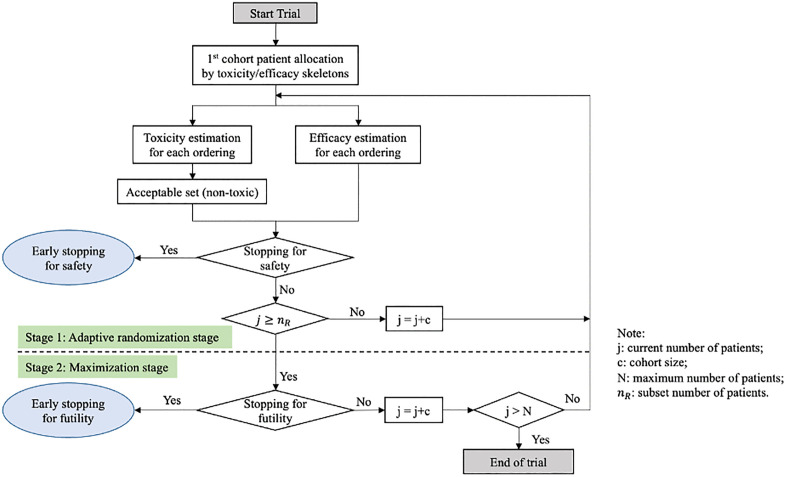
Flowchart of the general algorithm for one trial.

We extended the methodology by deriving various additional link functions and incorporating a wide range of prior distributions beyond the standard normal distribution, with customized parameter values. Additionally, we introduced the scale transformations tailored to the specific characteristics of each link function and prior distribution. (Fig 1) illustrates the general flowchart of this process.

In this section, we delved into the details of this algorithm. Specifically, the algorithm integrated diverse link functions such as hyperbolic tangent function, empiric function, one-parameter logistic function and two-parameter logistic function, making it compatible with a range of widely used prior distributions.

### Construction from partial orderings

Partial ordering followed the principle that both dose-toxicity and dose-efficacy curves for a single drug should monotonically increase, which helped determine the possible orderings for the drug combinations with different dosages. More specifically, for any single drug, a higher dose level should correspond to higher DLT and efficacy rates, assuming the dose levels of other drugs remain constant. However, the number of potential orderings can grow exponentially with the addition of more dose levels for a single drug or more drugs in the combination, making it impractical to explore every possible combination. Six typical orderings were suggested to manage this complexity from practical designs [34].

To illustrate this, two drugs A and B with I possible drug combinations $\;\{d_{\text{1}},d_{\text{2}}\ldots ,d_{I}\}$ were first considered, leading to M complete toxicity orders and K complete efficacy orders. We followed the example set by Wages and Conaway [23], which assumed three dose levels for each single drug, as shown in Table 1.

**Table 1:** 3×3 matrix for two drug combinations with different doses.

Dose level of A	Dose level of B		
	1	2	3
1	$d_{\text{1}}$	$d_{\text{2}}$	$d_{\text{3}}$
2	$d_{\text{4}}$	$d_{\text{5}}$	$d_{\text{6}}$
3	$d_{\text{7}}$	$d_{\text{8}}$	$d_{\text{9}}$

By holding the dose of one drug constant, the probabilities of toxicity and efficacy for the other dose increased monotonically across each row or column. For example, if we denoted the DLT probability at dose combination $d_{i}$ be $\pi_{T}\left(d_{i}\right)$, then the DLT probabilities followed $\pi_{T}\left(d_{\text{7}}\right)\leq \pi_{T}\left(d_{\text{8}}\right)\leq \pi_{T}\left(d_{\text{9}}\right)$ when dose A was set at level 3. Consequently, six complete toxicity orderings for the 9 dose combinations were outlined below. Similarly, six complete efficacy orderings can be established using analogous reasoning.

- Across rows: $\pi_{T}\left(d_{\text{1}}\right)\leq \pi_{T}\left(d_{\text{2}}\right)\leq \pi_{T}\left(d_{\text{3}}\right)\leq \pi_{T}\left(d_{\text{4}}\right)\leq \pi_{T}\left(d_{\text{5}}\right)\leq \pi_{T}\left(d_{\text{6}}\right)\leq \pi_{T}\left(d_{\text{7}}\right)\leq \pi_{T}\left(d_{\text{8}}\right)\leq \pi_{T}\left(d_{\text{9}}\right)$
- Up columns: $\pi_{T}\left(d_{\text{1}}\right)\leq \pi_{T}\left(d_{\text{4}}\right)\leq \pi_{T}\left(d_{\text{7}}\right)\leq \pi_{T}\left(d_{\text{2}}\right)\leq \pi_{T}\left(d_{\text{5}}\right)\leq \pi_{T}\left(d_{\text{8}}\right)\leq \pi_{T}\left(d_{\text{3}}\right)\leq \pi_{T}\left(d_{\text{6}}\right)\leq \pi_{T}\left(d_{\text{9}}\right)$
- Up diagonals: $\pi_{T}\left(d_{\text{1}}\right)\leq \pi_{T}\left(d_{\text{2}}\right)\leq \pi_{T}\left(d_{\text{4}}\right)\leq \pi_{T}\left(d_{\text{3}}\right)\leq \pi_{T}\left(d_{\text{5}}\right)\leq \pi_{T}\left(d_{\text{7}}\right)\leq \pi_{T}\left(d_{\text{6}}\right)\leq \pi_{T}\left(d_{\text{8}}\right)\leq \pi_{T}\left(d_{\text{9}}\right)$
- Down diagonals: $\pi_{T}\left(d_{\text{1}}\right)\leq \pi_{T}\left(d_{\text{4}}\right)\leq \pi_{T}\left(d_{\text{2}}\right)\leq \pi_{T}\left(d_{\text{7}}\right)\leq \pi_{T}\left(d_{\text{5}}\right)\leq \pi_{T}\left(d_{\text{3}}\right)\leq \pi_{T}\left(d_{\text{8}}\right)\leq \pi_{T}\left(d_{\text{6}}\right)\leq \pi_{T}\left(d_{\text{9}}\right)$
- Alternating down-up diagonals: $\pi_{T}\left(d_{\text{1}}\right)\leq \pi_{T}\left(d_{\text{2}}\right)\leq \pi_{T}\left(d_{\text{4}}\right)\leq \pi_{T}\left(d_{\text{7}}\right)\leq \pi_{T}\left(d_{\text{5}}\right)\leq \pi_{T}\left(d_{\text{3}}\right)\leq \pi_{T}\left(d_{\text{6}}\right)\leq \pi_{T}\left(d_{\text{8}}\right)\leq \pi_{T}\left(d_{\text{9}}\right)$
- Alternating up-down diagonals: $\pi_{T}\left(d_{\text{1}}\right)\leq \pi_{T}\left(d_{\text{4}}\right)\leq \pi_{T}\left(d_{\text{2}}\right)\leq \pi_{T}\left(d_{\text{3}}\right)\leq \pi_{T}\left(d_{\text{5}}\right)\leq \pi_{T}\left(d_{\text{7}}\right)\leq \pi_{T}\left(d_{\text{8}}\right)\leq \pi_{T}\left(d_{\text{6}}\right)\leq \pi_{T}\left(d_{\text{9}}\right)$

### Phase I/II CRM dose-finding design for drug combinations

A random variable $X_{j}$ denoted the drug combination for the j th patient, where $x_{j}\in \{d_{\text{1}},\ldots ,d_{I}\}$. Consider toxicity and efficacy as binary endpoints, so that for patient j,


$$Y_{j}=\left\{ \begin{array}{cc} \text{0}, & if\;no\;toxicity,\\ \text{1}, & if\;toxicity, \end{array}\right.\;{Z_{j}}_{j}=\left\{ \begin{array}{cc} \text{0}, & if\;no\;response,\\ \text{1}, & if\;response. \end{array}\right.\;$$

For I drug combinations, the pre-specified standardized unit, named toxicity skeleton, was used in the dose-toxicity curve to estimate the true toxicity probability $\pi_{T}\left(d_{i}\right)$. The toxicity skeleton had a length of I, denoted by $\text{0}<p_{\text{1}m}<p_{\text{2}m}<\cdots <p_{Im}<\text{1}$, where the ith toxicity skeleton for toxicity ordering m (denoted by $p_{im}$) was assigned to the drug combination with ith toxicity probability arranged in ascending order for the specific toxicity ordering m, across a total of M toxicity orderings. It is worth noting that while the I pre-specified toxicity skeleton remained the same, it was assigned to drug combinations with different orders corresponding to the toxicity orderings. These orderings had pre-determined prior probabilities τ(m)∈{τ(1),…,τ(M)}, as outlined in the section about partial ordering, and satisfied the conditions that $\sum_{m}\tau (m)=\text{1}$ and τ(m)≥0. Similarly, the pre-specified efficacy skeleton for I drug combinations, $\text{0}<q_{\text{1}k}<q_{\text{2}k}<\cdots <q_{Ik}<\text{1}$, had each efficacy ordering k linked to a skeleton ordering, across K efficacy orderings. These orderings came with prior probabilities ξ(k)∈{ξ(1),…,ξ(K)}, which satisfy the conditions that $\sum_{k}\xi (k)=\text{1}$ and ξ(k)≥0. In case where there was no prior information on orderings, a discrete uniform distribution could be employed.

### Starting the trial

The trial initiated by selecting the orderings of toxicity $m^{*}$ and efficacy $k^{*}$ with maximum prior probabilities in τ(m) and ξ(k). If multiple orderings shared the same maximum prior probability, one ordering was chosen at random. With the initial dose combinations $x_{\text{1}}$ chosen from $\{d_{\text{1}},\ldots ,d_{I}\}$, and the pre-specified toxicity skeleton $p_{im^{*}}$, the acceptable set was then defined as


$$A_{\text{1}}=\{d_{i}:\;p_{im^{*}}\leq \varphi_{T},i=\text{1},\;\ldots ,I\},$$

where $\phi_{T}$ was the pre-specified maximum acceptable toxicity rate (toxicity threshold). Subsequently, the randomization ratio was calculated as


$$R_{i}=\frac{q_{ik^{*}}}{\sum_{d_{i}\in A_{\text{1}}}q_{ik^{*}}}$$

in which $q_{ik^{*}}$ represented the pre-specified efficacy skeleton for the efficacy ordering $k^{*}$. The first patient cohort was randomized to combination $x_{\text{1}}=d_{i}$ with probability $R_{i}$.

### Toxicity estimation

Based on the partial orderings discussed before, the DLT probability for each ordering m was expressed as: $\pi_{T}\left(d_{i}\right)=P\left[Y_{j}=\text{1}|d_{i}\right]\approx F_{m}\left(d_{i},\beta \right),\;m=\text{1},\ldots ,M$,

where $F_{m}\left(d_{i},\beta \right)$ denoted the dose-toxicity model, often called the link function.

Upon enrolling j patients, the collected toxicity data were represented by $T_{j}=\{\left(x_{\text{1}},y_{\text{1}}\right),\ldots ,\left(x_{j},y_{j}\right)\}$. For each ordering m, the estimate of parameter β, ${\hat{\beta }}_{jm}$, was determined through Bayesian inference. In **crm12Comb**, the process for updating the likelihood function drew inspiration from the method used in the R package **dfcrm** [27] and was extended to include a variety of link functions with different prior distributions. Briefly, the prior distribution of β was assumed to be g(β), and τ(m) represented the prior distribution for the ordering m. The likelihood function was then formulated as:


$$L_{m}\left(\beta |T_{j}\right)=\prod_{l=\text{1}}^{j}\{F_{m}\left(x_{l},\beta \right){\}}^{y_{l}}\{\text{1}-F_{m}\left(x_{l},\beta \right){\}}^{\text{1}-y_{l}}$$ (1)

From this, the posterior density for β was defined as:


$$P_{m}\left(\beta |T_{j}\right)=\frac{L_{m}\left(\beta |T_{j}\right)g(\beta )}{\int_{B}L_{m}\left(\beta |T_{j}\right)g(\beta )d\beta },$$ (2)

and posterior density for ordering m became:


$$w\left(m|T_{j}\right)=\frac{\tau (m)\int_{B}L_{m}\left(\beta |T_{j}\right)g(\beta )d\beta }{\sum_{m=\text{1}}^{M}\tau (m)\int_{B}L_{m}\left(\beta |T_{j}\right)g(\beta )d\beta }$$ (3)

After each new patient or cohort of patients were enrolled, the probabilities of ordering τ(m) were sequentially updated using the posterior density in Equation (3). The ordering $m^{*}$ with the largest posterior probability was chosen according to:


$$m^{*}=\text{arg}\underset{m}{\text{max}}w\left(m|T_{j}\right),\;m=\text{1},\ldots ,M$$

Then, the posterior probability of DLT was obtained by:


$$\hat{\pi_{T}}\left(d_{im^{*}}\right)=F_{m^{*}}\left(d_{i},{\hat{\beta }}_{jm^{*}}\right),\;{\hat{\beta }}_{jm^{*}}=\int_{B}\beta P_{m^{*}}\left(\beta |T_{j}\right)d$$

Based on this, the acceptable set were defined as $A_{j}=\{d_{i}:\;\hat{\pi_{T}}\left(d_{im^{*}}\right)\leq \varphi_{T},\;i=\text{1},\ldots ,I\}$. If at any point during the trial the acceptable set became empty, the next patient would be allocated to the lowest dose level, $d_{\text{1}}$.

### Efficacy estimation

The method for efficacy estimation was similar to the approach used for toxicity estimation. Let K be the number of possible efficacy orderings, the efficacy probability for each ordering k was then expressed as: $\pi_{E}\left(d_{i}\right)=P\left(Z_{j}=\text{1}|d_{i}\right)\approx F_{k}\left(d_{i},\theta \right),\;k=\text{1},\ldots ,K$.

Utilizing Bayesian inference, the likelihood function of order k, based on the efficacy data collected from currently enrolled patients $E_{j}=\{\left(x_{\text{1}},z_{\text{1}}\right),\ldots ,\left(x_{j},z_{j}\right)\}$, was defined as:

$L_{k}\left(\theta |E_{j}\right)=\prod_{l=\text{1}}^{j}\{F_{k}\left(x_{l},\theta \right){\}}^{z_{l}}\{\text{1}-F_{k}\left(x_{l},\theta \right){\}}^{\text{1}-z_{l}}$

Then the posterior probabilities for parameter θ and ordering k were determined by:


$$P_{k}\left(\theta |E_{j}\right)=\frac{L_{k}\left(\theta |E_{j}\right)h(\theta )}{\int_{\Theta }L_{k}\left(\theta |E_{j}\right)h(\theta )d\theta }$$

$$w\left(k|E_{j}\right)=\frac{\xi (k)\int_{\Theta }L_{k}\left(\theta |E_{j}\right)h(\theta )d\theta }{\sum_{k=\text{1}}^{K}\xi (k)\int_{\Theta }L_{k}\left(\theta |E_{j}\right)h(\theta )d\theta }$$

From these, the estimate for θ, ${\hat{\theta }}_{jk}$, was obtained. For subsequent patient or cohort of patients, the order $k^{*}$ with the highest posterior probability was selected, and the efficacy probability for each drug combination under ordering $k^{*}$ was estimated as:


$$\hat{\pi_{E}}\left(d_{ik^{*}}\right)=F_{k^{*}}\left(d_{i},{\hat{\theta }}_{jk^{*}}\right),\;{\hat{\theta }}_{jk^{*}}=\int_{\Theta }\theta P_{k^{*}}\left(\theta |E_{j}\right)d,$$

Then $\hat{\pi_{E}}\left(d_{ik^{*}}\right)$ was used to guide the allocation of new patient or patient cohort.

### Patient allocation

To ensure each patient was allocated to the drug combination with the highest efficacy within the acceptable toxicity set, a two-phase allocation algorithm was implemented: an adaptive randomization phase for the initial stage with limited data, followed by a maximization phase when sufficient data are available.

The adaptive randomization phase aimed to prevent the trial from being stuck to the initially selected drug combinations, which allowed for exploration of a broader range of combinations. The randomization ratio $R_{i}$ was calculated as:


$$R_{i}=\frac{\hat{\pi_{E}}\left(d_{ik^{*}}\right)}{\sum_{d_{i}\in A_{j}}\hat{\pi_{E}}\left(d_{ik^{*}}\right)}$$ (4)

The next patient or cohort of patients was then randomized to combination $d_{i}$ with probability $R_{i}$, for $d_{i}\in A_{j}$. This randomization phase was applied to a pre-specified subset of $n_{R}$ patients.

Upon exceeding the $n_{R}$ patient threshold, the trial transitioned to the maximization phase, where patients were allocated to the combination $x_{j+\text{1}}$ that maximized estimated efficacy probability, determined by:


$$x_{j+\text{1}}=\text{arg}\underset{d_{i}\in A_{j}}{\text{max}}\hat{\pi_{E}}\left(d_{ik^{*}}\right)$$

The trial stopped once the total number of enrolled patients reached the pre-determined maximum of N, or when pre-defined stopping rules were met. At this point, the optimal dose combination (ODC) was defined as $d_{i}=x_{N+\text{1}}$ after the maximum sample size N patients have been enrolled. If the trial stopped earlier, no ODC was identified. We will introduce the details about early stopping rules in the following subsection.

### Early stopping rules

The trial incorporated stopping rules for both safety concerns and lack of efficacy (futility). At any stage of the trial, the exact binomial 95% confidence interval for toxicity at the least toxic combination $d_{\text{1}}$ was computed as $\left[\pi_{T}^{-}\left(d_{\text{1}}\right),\pi_{T}^{+}\left(d_{\text{1}}\right)\right]$. If the lower bound $\pi_{T}^{-}\left(d_{\text{1}}\right)$ exceeded the maximum acceptable toxicity rate $\varphi_{T}$, the trial stopped for safety, and no drug combination was designated as ODC.

Furthermore, the trial could also be stopped for futility if there were evidences suggesting no new dose combination had better effect than the existing ones in terms of efficacy. Upon enrolling j patients, the exact binomial 95% CI for efficacy at current combination $x_{j}$ was computed as $\left[\pi_{E}^{-}\left(x_{jk^{*}}\right),\pi_{E}^{+}\left(x_{jk^{*}}\right)\right]$. If the upper bound $\pi_{E}^{+}\left(x_{jk^{*}}\right)$ fell below the pre-specified futility threshold $\varphi_{E}$, the trial stopped for futility, and no drug combination was designated as ODC. It is important to note that the futility-based stopping rule was only applicable in the maximization phase, which started after enrolling $j\geq n_{R}$ patients, since the upper bound was derived from the maximum estimated efficacy probability during this phase.

### The implementation of link functions

The algorithm proposed by Wages and Conaway [23] in prior section utilized an empiric link function with a standard normal prior distribution to represent both the true probabilities of toxicity (under ordering m) and efficacy (under ordering k) as follows:


$$F_{m}\left(d_{i},\beta \right)=p_{im}^{\text{exp}(\beta )},\beta \in B=(-\infty ,\infty )\;F_{k}\left(d_{i},\theta \right)=q_{ik}^{\text{exp}(\theta )},\theta \in \Theta =(-\infty ,\infty ),$$

with β and θ assuming standard normal prior distributions having expectation at 0, i.e., E[β]=0 and E[θ]=0. This approach, favoring the exponential form over a linear one, accounted for the presumed monotonic nature of the dose-toxicity and dose-efficacy curves.

Expanding on this framework, this section introduces a broader range of prior distributions for β and θ, allowing for more versatile selections. The detailed derivation of two-parameter link functions is provided in the S1 File. Additionally, the corresponding scale transformation of the skeleton was also applied, following the methodology outlined in [4], to enhance the model’s adaptability. Since the above prior distribution of empiric link function followed N(0,1), no scale transformation is needed. The details are given in Table 2.

**Table 2:** Link functions for toxicity and efficacy probabilities with scale transformations and prior distributions.

Name	Type	Link functions	Prior distribution	Scale transformation
Empiric	Toxicity	$F_{m}\left(d_{i},\beta \right)={\tilde{p}}_{im}^{exp(\beta )}$	Normal: $\beta \sim N\left(\mu_{\beta },\sigma_{\beta }^{\text{2}}\right)$ Gamma: $\beta \sim Gamma\left(a_{\beta },b_{\beta }\right)$	${\tilde{p}}_{im}=p_{im}^{\text{exp}\left(-\mu_{\beta }\right)}$ ${\tilde{p}}_{im}=p_{im}^{\left(b_{\beta }/a_{\beta }\right)}$
	Efficacy	$F_{k}\left(d_{i},\theta \right)={\tilde{q}}_{ik}^{\text{exp}(\theta )}$	Normal: $\theta \sim N\left(\mu_{\theta },\sigma_{\theta }^{\text{2}}\right)$ Gamma: $\theta \sim Gamma\left(a_{\theta },b_{\theta }\right)$	${\tilde{q}}_{ik}=q_{ik}^{\text{exp}\left(-\mu_{\theta }\right)}$ ${\tilde{q}}_{ik}=q_{ik}^{\left(b_{\theta }/a_{\theta }\right)}$
Hyperbolic tangent	Toxicity	$F_{m}\left(d_{i},\beta \right)={\left(\frac{\text{tanh}\left({\tilde{p}}_{im}\right)+\text{1}}{\text{2}}\right)}^{\beta }$	Exponential: $\beta \sim Exp\left(\lambda_{\beta }\right)$	${\tilde{p}}_{im}=\text{arctanh}\left(\text{2}\times p_{im}^{\frac{\text{1}}{\lambda_{\beta }}}-\text{1}\right)$
	Efficacy	$F_{k}\left(d_{i},\theta \right)={\left(\frac{\text{tanh}\left({\tilde{q}}_{ik}\right)+\text{1}}{\text{2}}\right)}^{\theta }$	Exponential: $\theta \sim Exp\left(\lambda_{\theta }\right)$	${\tilde{q}}_{ik}=\text{arctanh}\left(\text{2}\times q_{ik}^{\frac{\text{1}}{\lambda_{\theta }}}-\text{1}\right)$
One-parameter logistic	Toxicity	Normal: $F_{m}\left(d_{i},\beta \right)=\frac{\text{1}}{\text{1}+\text{exp}\left(-a_{\text{0}T}-\text{exp}(\beta ){\tilde{p}}_{im}\right)}$ Gamma: $F_{m}\left(d_{i},\beta \right)=\frac{\text{1}}{\text{1}+\text{exp}\left(-a_{\text{0}T}-\beta {\tilde{p}}_{im}\right)}$	Normal: $\beta \sim N\left(\mu_{\beta },\sigma_{\beta }^{\text{2}}\right)$ Gamma: $\beta \sim Gamma\left(a_{\beta },b_{\beta }\right)$	${\tilde{p}}_{im}=\frac{\text{log}\left(\frac{p_{im}}{\text{1}-p_{im}}\right)-a_{\text{0}T}}{\text{exp}\left(\mu_{\beta }\right)}$ ${\tilde{p}}_{im}=\frac{\text{log}\left(\frac{p_{im}}{\text{1}-p_{im}}\right)-a_{\text{0}T}}{\left(a_{\beta }/b_{\beta }\right)}$
	Efficacy	Normal: $F_{k}\left(d_{i},\theta \right)=\frac{\text{1}}{\text{1}+\text{exp}\left(-a_{\text{0}E}-\text{exp}(\theta ){\tilde{q}}_{ik}\right)}$ Gamma: $F_{k}\left(d_{i},\theta \right)=\frac{\text{1}}{\text{1}+\text{exp}\left(-a_{\text{0}E}-\theta {\tilde{q}}_{ik}\right)}$	Normal: $\theta \sim N\left(\mu_{\theta },\sigma_{\theta }^{\text{2}}\right)$ Gamma: $\theta \sim Gamma\left(a_{\theta },b_{\theta }\right)$	${\tilde{q}}_{ik}=\frac{\text{log}\left(\frac{q_{ik}}{\text{1}-q_{ik}}\right)-a_{\text{0}E}}{\text{exp}\left(\mu_{\theta }\right)}$ ${\tilde{q}}_{ik}=\frac{\text{log}\left(\frac{q_{ik}}{\text{1}-q_{ik}}\right)-a_{\text{0}E}}{\left(a_{\theta }/b_{\theta }\right)}$
Two-parameter logistic	Toxicity	Normal: $F_{m}\left(d_{i},\beta \right)=\frac{\text{1}}{\text{1}+\text{exp}\left(-\alpha_{T}-\text{exp}(\beta ){\tilde{p}}_{im}\right)}$ Gamma: $F_{m}\left(d_{i},\beta \right)=\frac{\text{1}}{\text{1}+\text{exp}\left(-\alpha_{T}-\beta {\tilde{p}}_{im}\right)}$	Normal: $\alpha_{T}\sim N\left(\mu_{\alpha_{T}},\sigma_{\alpha_{T}}^{\text{2}}\right)$ $\beta \sim N\left(\mu_{\beta },\sigma_{\beta }^{\text{2}}\right)$ Gamma: $\alpha_{T}\sim Gamma\left(a_{\alpha_{T}},b_{\alpha_{T}}\right)$ $\beta \sim Gamma\left(a_{\beta },b_{\beta }\right)$	${\tilde{p}}_{im}=\frac{\text{log}\left(\frac{p_{im}}{\text{1}-p_{im}}\right)-\mu_{\alpha_{T}}}{\text{exp}\left(\mu_{\beta }\right)}$ ${\tilde{p}}_{im}=\frac{\text{log}\left(\frac{p_{im}}{\text{1}-p_{im}}\right)-\frac{a_{\alpha_{T}}}{b_{\alpha_{T}}}}{\left(a_{\beta }/b_{\beta }\right)}$
	Efficacy	Normal: $F_{k}\left(d_{i},\theta \right)=\frac{\text{1}}{\text{1}+\text{exp}\left(-\alpha_{E}-\text{exp}(\theta ){\tilde{q}}_{ik}\right)}$ Gamma: $F_{k}\left(d_{i},\theta \right)=\frac{\text{1}}{\text{1}+\text{exp}\left(-\alpha_{E}-\theta {\tilde{q}}_{ik}\right)}$	Normal: $\alpha_{E}\sim N\left(\mu_{\alpha_{E}},\sigma_{\alpha_{E}}^{\text{2}}\right)$ $\theta \sim N\left(\mu_{\theta },\sigma_{\theta }^{\text{2}}\right)$ Gamma: $\alpha_{E}\sim Gamma\left(a_{\alpha_{E}},b_{\alpha_{E}}\right)$ $\theta \sim Gamma\left(a_{\theta },b_{\theta }\right)$	${\tilde{q}}_{ik}=\frac{\text{log}\left(\frac{q_{ik}}{\text{1}-q_{ik}}\right)-\mu_{\alpha_{E}}}{\text{exp}\left(\mu_{\theta }\right)}$ ${\tilde{q}}_{ik}=\frac{\text{log}\left(\frac{q_{ik}}{\text{1}-q_{ik}}\right)-\frac{a_{\alpha_{E}}}{b_{\alpha_{E}}}}{\left(a_{\theta }/b_{\theta }\right)}$

Note: $d_{i}$ denotes the ith drug combination, $p_{im}$ denotes the pre-specified toxicity skeleton for ith drug combination and mth toxicity ordering, and $q_{ik}$ denotes the pre-specified efficacy skeleton for ith drug combination and kth efficacy ordering.

### Using crm12Comb

The R package **crm12Comb** contains functions that can perform simulations for integrated Phase I and II adaptive design for drug combinations using the CRM dose-finding design. It offers features to generate skeletons and specifications for different link functions, enabling users to conduct comprehensive simulation studies with pre-specified information to assess operating characteristics, or to determine the appropriate dose combination for upcoming patient or patient cohort based on all accumulated data.

To begin, we need to first install the **crm12Comb** package, and then load it as shown below:

**Table:** 

install.packages(“crm12Comb”) library(crm12Comb)

Following that, in the section below we will provide examples on how to use the **crm12Comb** to simulate multiple trials to obtain operating characteristics of phase I/II adaptive design for drug combinations using CRM. Additionally, we will demonstrate how to simulate a single trial for the allocation of the next patient or patient cohort based on the most recent cumulated data. Moreover, we will illustrate an example code to perform multiple simulations with different sets of inputs for trial settings.

### Simulation of multiple trials

Using a 4 ×4 dose combination for two drugs as an example, the levels of dose combinations can be obtained based on the function doseComb_to_mat(),

**Table:** 

doseComb_to_mat(c(4,4), type = “matrix”) [, 1] [, 2] [, 3] [, 4] [1,] 1 2 3 4 [2,] 5 6 7 8 [3,] 9 10 11 12 [4,] 13 14 15 16

Then, the pre-defined true probabilities of toxicity and efficacy related to the two-drug combinations can be input sequentially, corresponding to the data presented in Table 3. Other values need to be pre-specified include 1000 simulation trials, maximum sample size as 40, subset number of patients as 20, toxicity threshold $\varphi_{T}=\text{3}\text{0}\%$, efficacy threshold is 30%, cohort size as 1 patient, correlation between toxicity and efficacy binary endpoints as 0 indicating independence between toxicity and efficacy, and empiric link function with normal prior distribution (mean 0 and standard deviation 1) for both toxicity and efficacy probabilities.

**Table 3:** Example of true (toxicity, efficacy) probabilities for a 4 ×4 combinations.

Doses of A	Doses of B			
	1	2	3	4
1	(0.02,0.05)	(0.04,0.10)	(0.08,0.15)	**(0.12,0.32)**
2	(0.06,0.10)	(0.10,0.15)	(0.14,0.25)	**(0.20,0.35)**
3	(0.12,0.18)	(0.16,0.22)	**(0.22,0.35)**	**(0.25,0.40)**
4	(0.20,0.24)	**(0.24,0.35)**	(0.35,0.45)	(0.40,0.50)

Target combinations are denoted in bold type when satisfying both acceptable toxicity (≤30%) and high efficacy (≥30%).

**Table:** 

scenario < - matrix(c(0.02, 0.05, 0.04, 0.10, 0.08, 0.15, 0.12, 0.32, 0.06, 0.10, 0.10, 0.15, 0.14, 0.25, 0.20, 0.35, 0.12, 0.18, 0.16, 0.22, 0.22, 0.35, 0.25, 0.40, 0.20, 0.24, 0.24, 0.35, 0.35, 0.45, 0.40, 0.50), ncol = 2, byrow = TRUE)

The toxicity and efficacy skeletons can be constructed by either using prior information from historical data or by employing the algorithm proposed by Lee and Cheung [35]. The getprior() function from the R package **dfcrm** [27], which implemented the algorithm of the empiric model and one-parameter logistic model, assuming a normal prior distribution of the parameter with a mean of zero. We adapted the getprior() function in **dfcrm** to build the priorSkeletons() function in our **crm12Comb**, making it compatible with the link functions and prior distributions in the methods section. This adaptation allows for user-defined parameters for various distributions. The toxicity and efficacy skeletons are generated using the priorSkeletons() function, and we followed the same default settings of getprior() function [27] in this example.

**Table:** 

DLT_skeleton < - priorSkeletons(updelta = 0.025, target = 0.3, npos = 10, ndose = 16, model = “logistic”, prior = “normal”, beta_mean = 0) > DLT_skeleton [1] 0.02366005 0.03501856 0.05036881 0.07043821 [5] 0.09580864 0.12680087 0.16337339 0.20506526 [9] 0.25100622 0.30000000 0.35066402 0.40159193 [13] 0.45150111 0.49933748 0.54432656 0.58597596 Efficacy_skeleton < - priorSkeletons(updelta = 0.025, target = 0.5, npos = 10, ndose = 16, model = “logistic”, prior = “normal”, beta_mean = 0) > Efficacy_skeleton [1] 0.07796568 0.10744537 0.14347745 0.18578508 [4] 0.23352357 0.28535019 0.33959663 0.39449801 [9] 0.44841540 0.50000000 0.54827532 0.59264300 [13] 0.63283541 0.66884237 0.70083351 0.72908986 [11] 0.54883378 0.59493664 0.63795661 0.67769651

The steps above are the preparations for the simulations. Next, we can run simulations using the SIM_phase_I_II() function, which outputs 9 operating characteristics. These include:

(1) the probability of recommending safe/ineffective combinations as ODC ($prob_safe in R output),
(2) the probability of recommending target combinations as ODC ($prob_target in R output),
(3) the probability of recommending toxic combinations as ODC ($prob_toxic in R output),
(4) the average number of patients enrolled ($mean_SS in R output),
(5) the average proportion of patients allocated to target ODS(s) ($mean_ODC in R output),
(6) the probability of simulation trials stopped early for safety ($prob_stop_safety in R output),
(7) the probability of simulation trials stopped early for futility ($prob_stop_futility in R output),
(8) the average observed DLT rate ($mean_DLT in R output),
(9) the average observed response rate ($mean_ORR in R output).

Besides the 9 operating characteristics, we also store the data of each trial into a list through the number of simulations ($datALL in R output). Since the DLT and efficacy data are randomly generated and the adaptive randomization phase involves randomly selecting the next allocation combination based on the randomization ratio in Equation (4), results may vary slightly across different selections of random seeds. Therefore, we recommend conducting a minimum of 1000 simulations to ensure the reliability of the results.

The SIM_phase_I_II() function includes the following arguments: nsim for number of simulation trials, Nmax for number of maximum enrolled patients for each trial, DoseComb for pre-defined true toxicity and efficacy probabilities for each drug combinations, input_doseComb_forMat for drug combinations, input_type_forMat for format of drug combinations, input_Nphase for number of subset patients, input_DLT_skeleton for pre-specified toxicity skeleton, input_efficacy_skeleton for pre-specified efficacy skeleton, input_DLT_thresh for DLT threshold to define acceptable set with default value 0.3, input_efficacy_thresh for efficacy threshold to define target combinations with default value 0.3, input_M_prob for prior probabilities of toxicity orderings (sum is 1), input_K_prob for prior probabilities of efficacy orderings (sum is 1), input_cohortsize for number of patients in each cohort, input_corr for association parameter for efficacy and toxicity (0 means independent), input_early_stopping_safety_thresh for safety threshold for early stopping with default value 0.33, input_early_stopping_futility_thresh for futility threshold for early stopping with default value 0.2, input_model for the model of link function with default model "empiric", input_para_prior for the prior distribution of parameters in link function with default "normal", and other arguments are to specify the values of each parameter based on the prior distribution. For detailed description, run ?SIM_phase_I_II to check the documentation.

**Table:** 

simRes < - SIM_phase_I_II(nsim = 1000, Nmax = 40, DoseComb = scenario, input_doseComb_forMat = c(4,4), input_type_forMat = “matrix”, input_Nphase = 20, input_DLT_skeleton = DLT_skeleton, input_efficacy_skeleton = Efficacy_skeleton, input_DLT_thresh = 0.3, input_efficacy_thresh = 0.3, input_cohortsize = 1, input_corr = 0, input_early_stopping_safety_thresh = 0.33, input_early_stopping_futility_thresh = 0.2, input_model=“logistic”, input_para_prior=”normal”, input_beta_mean = 0, input_beta_sd = 1, input_theta_mean = 0, input_theta_sd = 1, random_seed = 23) > simRes$prob_safe [1] 0.002 > simRes$prob_target [1] 0.961 > simRes$prob_toxic [1] 0.034 > simRes$mean_SS [1] 39.94 > simRes$mean_ODC [1] 0.719 > simRes$prob_stop_safety [1] 0 > simRes$prob_stop_futility [1] 0.003 > simRes$mean_DLT [1] 0.191 > simRes$mean_ORR [1] 0.329

In addition, the dataset, including columns for dose combination, DLT outcome, and efficacy outcome for each simulation trial, is stored in a list, which can be viewed through simRes$datALL. In this example of 1000 simulations, the simRes$datALL is a list containing 1000 elements, each representing patient data enrolled sequentially. This command can also allow for the examination of detailed patient allocations, where efficacy outcome is denoted by ORR. For example, if we output the first 5 enrolled patients ([1:5,]) from the first simulated trial ($datALL[[1]]), the first patient is allocated to drug combination #5 without having any DLT or ORR outcomes based on the first row in the output below.

**Table:** 

> simRes$datALL[ [1]][1:5,] DoseLevel DLT ORR 1 5 0 0 2 4 0 1 3 10 0 0 4 12 1 1 5 10 0 0

**Fig 2:**
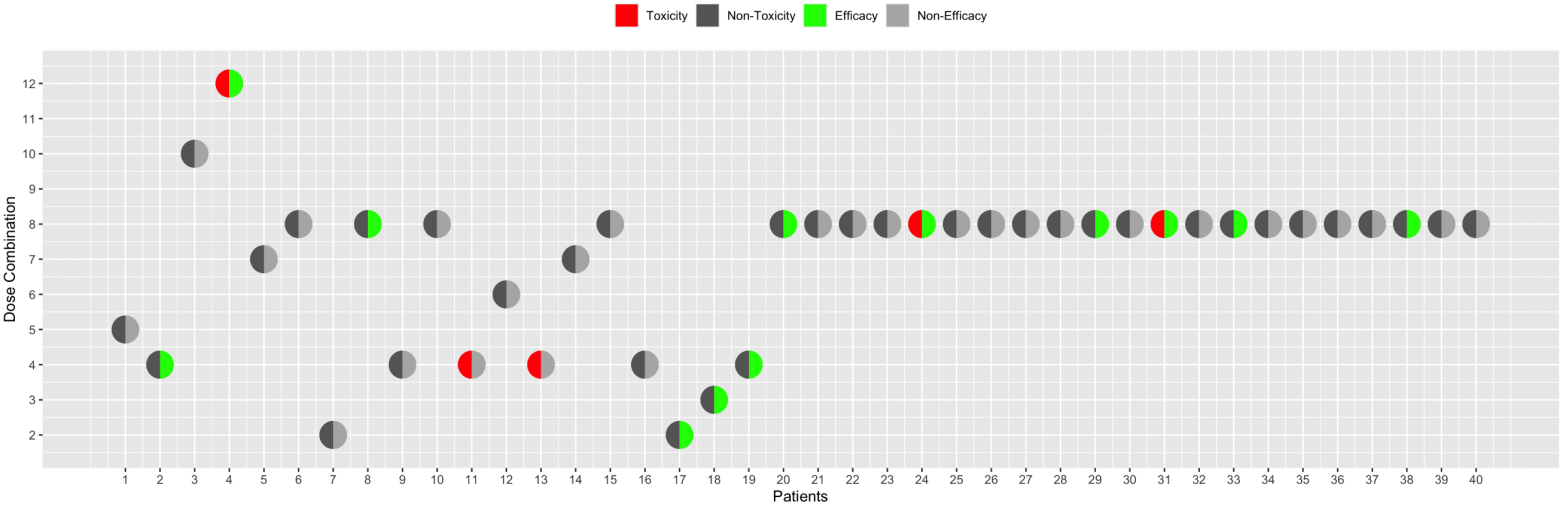
Patient enrolment with toxicity and efficacy outcomes for the first trial in simulation results 𝐬𝐢𝐦𝐑𝐞𝐬.

For visualization, three types of plots can be generated. To show the sequential enrolment of patients for a single trial, the enroll_patient_plot() function can be utilized. For example, to visualize the patient entry of the first simulated trial from the results simRes, we can use the syntax below to generate Fig 2, where the x-axis denotes the sequential order of patient enrolments and the y-axis represents the assigned dose combination for each patient. Patients experiencing DLT outcomes are indicated by a red-left half dot, those with efficacy outcomes by a green-right half dot, and patients without such outcomes are represented by grey dots.

outcomes are represented by grey dots.

**Table:** 

enroll_patient_plot(simRes$datALL[ [1]])

**Fig 3:**
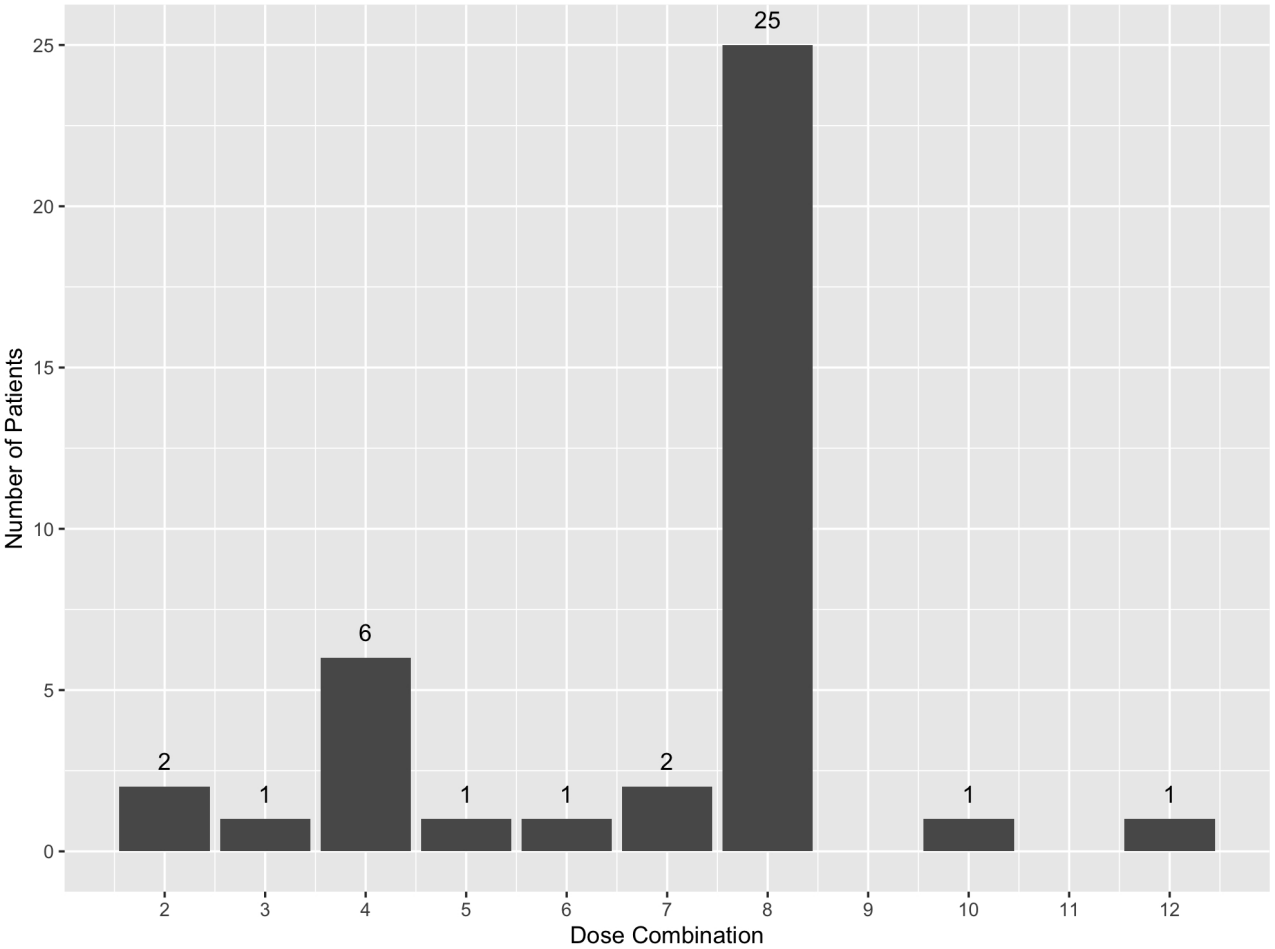
Patient allocations by dose combinations for the first trial in simulation results 𝐬𝐢𝐦𝐑𝐞𝐬.

The second plot, a histogram, displays the distribution of patients across each dose combination in a single simulated trial, which can be generated using the syntax patient_allocation_plot() funtcion. To visualize the patient allocations of the first simulated trial from the results simRes, we can use the syntax below to generate Fig 3 with specific number of patients allocated to each dose combination indicated at the top of each bar.

**Table:** 

patient_allocation_plot(simRes$datALL[ [1]])

**Fig 4:**
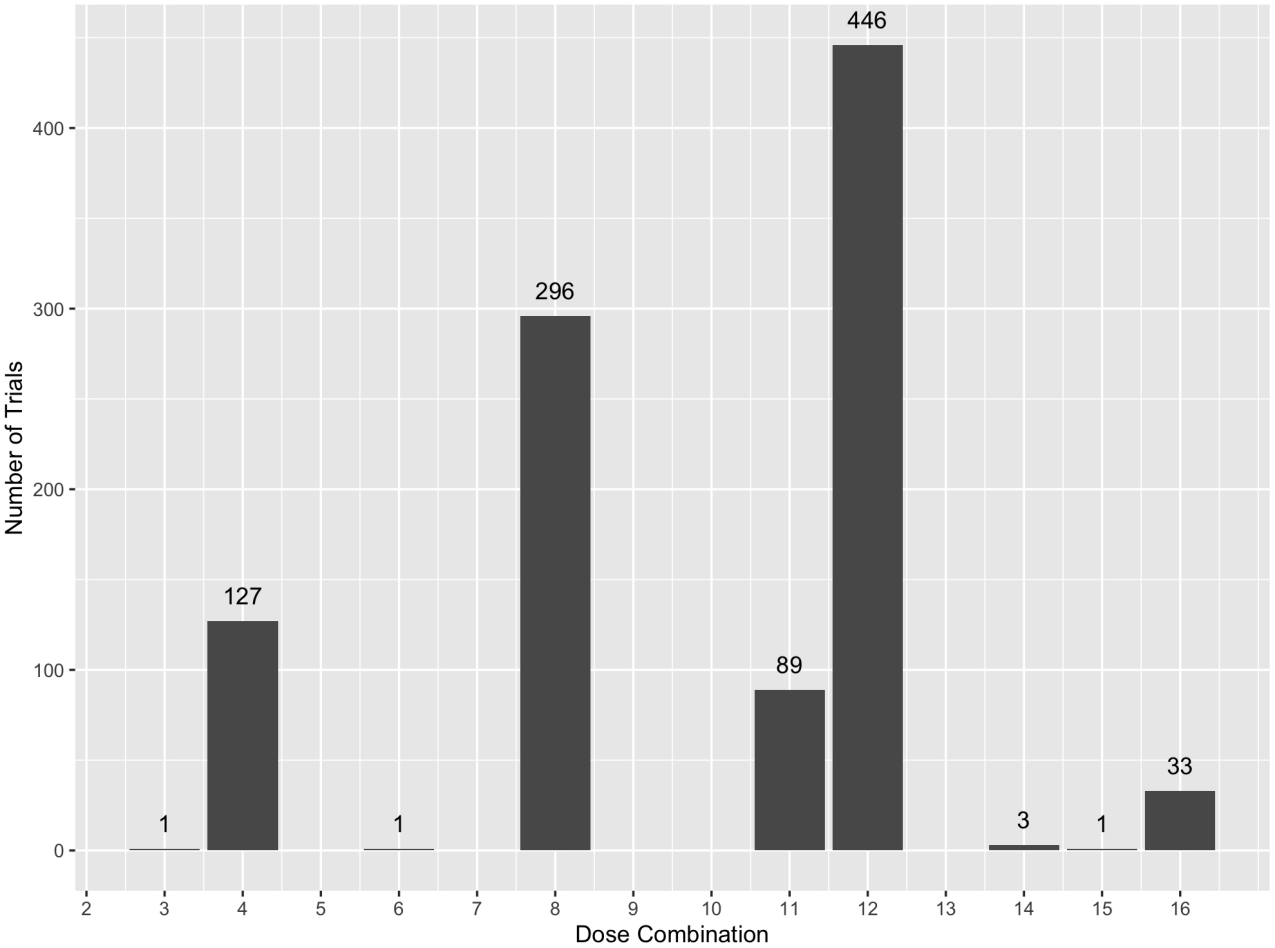
Dose combinations identified as ODC among all simulations in 𝐬𝐢𝐦𝐑𝐞𝐬.

The third plot is also a histogram that represents the number of trials in which each dose combination was identified as ODC. The plot can be created using the syntax ODC_plot() function. For the 1000 simulation trials contained in simRes, we can use the syntax below to generate Fig 4. This figure includes the specific counts of trials where each dose combination was deemed the ODC, indicated at the top of each bar. It is worth noting that among 1000 simulation trials, 4 trials with no ODC identified has been excluded from the figure because of stopping early for futility (simRes$prob_stop_futility=0.006).

**Table:** 

ODC_plot(simRes)

The outcomes related to operating characteristics may vary slightly by choosing different random seeds for the random nature of the following factors: (1) the toxicity and efficacy outcomes are randomly generated based on a bivariate binary correlation (which was set as 0 in the example above, input_corr=0), and (2) when multiple dose combinations have the same efficacy skeleton (when starting the trial and selecting dose combination for first patient allocation) and have the same maximum estimated efficacy probability (during the maximization phase), one dose combination will be chosen at random. Therefore, to ensure more reliable results while maintaining computational efficiency, conducting 1000 simulation runs is recommended.

### Simulation of a single trial patient allocation

Helper functions (such as get_orderings, toxicity_est, and efficacy_est) have been created to streamline the whole simulation process that is included in the SIM_phase_I_II() function, as described in this section. These functions can also be used to perform a single simulation to provide the allocation of the next patient based on the existing data.

An example dataset, representing six patients already enrolled using a 4x4 dose combination matrix, is provided below.

**Table:** 

> set.seed(23) > currDat < - data.frame(sample(1:6, 6, replace = TRUE), rbinom(6, 1, 0.2), rbinom(6, 1, 0.5)) > names(currDat) <- c(“DoseLevel”, “DLT”, “ORR”) > currDat DoseLevel DLT ORR 1 5 1 0 2 4 1 1 3 3 0 1 4 1 0 1 5 3 0 1 6 5 1 1

The get_ordering() function, as described in the section about partial ordering, can be implemented to get the possible orderings for dose combinations when there is no prior information available for the combined drugs. With prior knowledge, users can specify customized orderings by aggregating them into a list for the argument doseComb_forMat and set the argument type_forMat as "self" of the get_ordering() function.

**Table:** 

> orderings < - get_ordering(doseComb_forMat = c(4,4), type_forMat = “matrix”) > orderings [ [1]] [1] 1 2 3 4 5 6 7 8 9 10 11 12 13 14 15 16 [ [2]] [1] 1 5 9 13 2 6 10 14 3 7 11 15 4 8 12 16 [ [3]] [1] 1 5 2 9 6 3 13 10 7 4 14 11 8 15 12 16 [ [4]] [1] 1 2 5 3 6 9 4 7 10 13 8 11 14 12 15 16 [ [5]] [1] 1 5 2 3 6 9 13 10 7 4 8 11 14 15 12 16 [ [6]] [1] 1 2 5 9 6 3 4 7 10 13 14 11 8 12 15 16

From the orderings generated by the get_ordering() function, we can obtain the toxicity and efficacy orderings by modifying the orderings of the pre-specified skeletons, which were generated by the priorSkeletons() function described before. To condense the output, we only keep 3 digits in the example below (function with round(x,3)). However, in the actual calculation, we will use the original digits of each skeleton probability.

**Table:** 

> DLT < - lapply(orderings, function(or){DLT_skeleton[order(or)]}) > lapply(DLT, function (x) round(x, 3)) [ [1]] [1] 0.024 0.035 0.050 0.070 0.096 0.127 0.163 0.205 [9] 0.251 0.300 0.351 0.402 0.452 0.499 0.544 0.586 [ [2]] [1] 0.024 0.096 0.251 0.452 0.035 0.127 0.300 0.499 [9] 0.050 0.163 0.351 0.544 0.070 0.205 0.402 0.586 [ [3]] [1] 0.024 0.050 0.127 0.300 0.035 0.096 0.251 0.452 [9] 0.070 0.205 0.402 0.544 0.163 0.351 0.499 0.586
[ [4]] [1] 0.024 0.035 0.070 0.163 0.050 0.096 0.205 0.351 [9] 0.127 0.251 0.402 0.499 0.300 0.452 0.544 0.586 [ [5]] [1] 0.024 0.050 0.070 0.300 0.035 0.096 0.251 0.351 [9] 0.127 0.205 0.402 0.544 0.163 0.452 0.499 0.586 [ [6]] [1] 0.024 0.035 0.127 0.163 0.050 0.096 0.205 0.452 [9] 0.070 0.251 0.402 0.499 0.300 0.351 0.544 0.586 > ORR < - lapply(orderings, function(or){Efficacy_skeleton[order(or)]}) > lapply(ORR, function (x) round(x, 3)) [ [1]] [1] 0.078 0.107 0.143 0.186 0.234 0.285 0.340 0.394 [9] 0.448 0.500 0.548 0.593 0.633 0.669 0.701 0.729 [ [2]] [1] 0.078 0.234 0.448 0.633 0.107 0.285 0.500 0.669 [9] 0.143 0.340 0.548 0.701 0.186 0.394 0.593 0.729 [ [3]] [1] 0.078 0.143 0.285 0.500 0.107 0.234 0.448 0.633 [9] 0.186 0.394 0.593 0.701 0.340 0.548 0.669 0.729 [ [4]] [1] 0.078 0.107 0.186 0.340 0.143 0.234 0.394 0.548 [9] 0.285 0.448 0.593 0.669 0.500 0.633 0.701 0.729 [ [5]] [1] 0.078 0.143 0.186 0.500 0.107 0.234 0.448 0.548 [9] 0.285 0.394 0.593 0.701 0.340 0.633 0.669 0.729 [ [6]] [1] 0.078 0.107 0.285 0.340 0.143 0.234 0.394 0.633 [9] 0.186 0.448 0.593 0.669 0.500 0.548 0.701 0.729

Based on these established toxicity skeletons and orderings, the toxicity_est() function can be used to estimate toxicity probabilities for each dose combination, given current data. The function returns a list that contains dose combinations within the acceptable set and the posterior density of toxicity orderings, which are instrumental for subsequent toxicity estimations.

**Table:** 

> tox < - toxicity_est(Dat = currDat, I = 16, M = 6, M_prob = rep(1/6, 6), DLT_skeleton = DLT, DLT_thresh = 0.3, model = “empiric”, para_prior=”normal”, beta_mean = 0, beta_sd = 1, intcpt_lgst1 = NULL, beta_shape = NULL, beta_inverse_scale = NULL, alpha_mean = NULL, alpha_sd = NULL, alpha_shape = NULL, alpha_inverse_scale = NULL, seed = 23) > tox $mStar [1] 1 $piT_hat [1] 0.2874126 0.3275022 0.3696467 0.4133242 0.4579118 [6] 0.5027093 0.5469774 0.5899872 0.6310731 0.6696811 [11] 0.7054028 0.7379897 0.7673484 0.7935195 0.8166472 [16] 0.8369472 $AR [1] 1 $M_prob [1] 0.26095160 0.08809177 0.13373859 0.19276940 [5] 0.18463402 0.13981462

Following the determination of the acceptable set of dose combinations from the toxicity estimation (as indicated by the results below $AR), the efficacy_est() function can be used to estimate efficacy probabilities. This allows us to identify the most suitable dose combination for allocating the next patient or patient cohort.

**Table:** 

> eff < - efficacy_est(Dat = currDat, AR = tox$AR, I = 16, K = 6, K_prob = rep(1/6, 6), efficacy_skeleton = ORR, Nphas = 20, model = “empiric”, para_prior=”normal”, theta_mean = 0, theta_sd = 1, theta_intcpt_lgst1 = NULL, theta_shape = NULL, theta_inverse_scale = NULL, alphaT_mean = NULL, alphaT_sd = NULL, alphaT_shape = NULL, alphaT_inverse_scale = NULL, seed = 23, seed_rand = 23, seed_max = 23) > eff $kStar [1] 2 $piE_hat [1] 0.5163099 $di [1] 1 $K_prob [1] 0.09027006 0.26494238 0.19110582 0.13364874 [5] 0.15235978 0.16767323

The next patient or cohort of patients will be allocated to dose combination #1 and posterior density of efficacy orderings will be used for the next efficacy estimations.

### Examples of multiple simulations

To process multiple lists of input values for various parameters (such as N, $n_{R}$, corr, toxicity/efficacy skeletons, link functions, prior distributions, etc.), a for loop can be employed with the SIM_phase_I_II() function to obtain multiple sets of results. We adopted the scenario settings, including six scenarios with pre-defined true toxicity and true efficacy probabilities [23], as shown in Table 3. Here, for illustration, we further compare the results of three different link functions: empiric model with normal prior, hyperbolic tangent model with exponential prior, and one-parameter logistic model with normal and gamma priors. Besides, we also considered various combinations of two sets of toxicity and efficacy skeletons, the maximum number of patients per simulation trial (N∈(40,50,60)), correlation between toxicity and efficacy (corr∈(0,−2.049,0.814) as illustrated by Thall and Cook [36]), and the number of subset patients for choosing randomization or maximization phase ($n_{R}\in (\text{1}\text{0},\text{2}\text{0},\text{3}\text{0})$).

Using the empiric link function with a normal prior as an example, the code below demonstrates a method to derive results for a list of input values. First, we need to input the six scenarios in the form of 3×3 matrices. The examples of all six scenarios are shown below, with *i* th row denotes the toxicity and efficacy probabilities for the *i* th drug combination. doseComb_to_mat() shows the corresponding drug combinations linked to the positions in Table 4.

**Table 4:** Six scenarios of true (toxicity, efficacy) probabilities [23].

Scenario	Doses of A	True (toxicity, efficacy) probabilities		
		Doses of B		
		1	2	3
1	3	(0.08, 0.15)	(0.10, 0.20)	**(0.18, 0.40)**
	2	(0.04, 0.10)	(0.06, 0.16)	(0.08, 0.20)
	1	(0.02, 0.05)	(0.04, 0.10)	(0.06, 0.15)
2	3	(0.16, 0.20)	**(0.25, 0.35)**	(0.35, 0.50)
	2	(0.10, 0.10)	(0.14, 0.25)	**(0.20, 0.40)**
	1	(0.06, 0.05)	(0.08, 0.10)	(0.12, 0.20)
3	3	**(0.24, 0.40)**	(0.33, 0.50)	(0.40, 0.60)
	2	(0.16, 0.20)	**0.22, 0.40**	(0.35, 0.50)
	1	(0.08, 0.10)	(0.14, 0.25)	**(0.20, 0.35)**
4	3	(0.33, 0.50)	(0.40, 0.60)	(0.55, 0.70)
	2	**(0.18, 0.35)**	**(0.25, 0.45)**	(0.42, 0.55)
	1	(0.12, 0.20)	**(0.20, 0.40)**	(0.35, 0.50)
5	3	(0.45, 0.55)	(0.55, 0.65)	(0.75, 0.75)
	2	**(0.20, 0.36)**	(0.35, 0.49)	(0.40, 0.62)
	1	(0.15, 0.20)	**(0.20, 0.35)**	**(0.25, 0.50)**
6	3	(0.65, 0.60)	(0.80, 0.65)	(0.85, 0.70)
	2	(0.55, 0.55)	(0.70, 0.60)	(0.75, 0.65)
	1	(0.50, 0.50)	(0.55, 0.55)	(0.65, 0.60)
Combinations with acceptable toxicity (≤30%) and high efficacy (≥30%) are defined as target combinations shown in boldface type.				

**Table:** 

scenario1 < - matrix(c(0.02, 0.05, 0.04, 0.10, 0.06, 0.15, 0.04, 0.10, 0.06, 0.16, 0.08, 0.20, 0.08, 0.15, 0.10, 0.20, 0.18, 0.40 ), ncol = 2, byrow = TRUE) scenario2 < - matrix(c(0.06, 0.05, 0.08, 0.10, 0.12, 0.20, 0.10, 0.10, 0.14, 0.25, 0.20, 0.40, 0.16, 0.20, 0.25, 0.35, 0.35, 0.50 ), ncol = 2, byrow = TRUE) scenario3 < - matrix(c(0.08, 0.10, 0.14, 0.25, 0.20, 0.35, 0.16, 0.20, 0.22, 0.40, 0.35, 0.50, 0.24, 0.40, 0.33, 0.50, 0.40, 0.60 ), ncol = 2, byrow = TRUE) scenario4 < - matrix(c(0.12, 0.20, 0.20, 0.40, 0.35, 0.50, 0.18, 0.35, 0.25, 0.45, 0.42, 0.55, 0.33, 0.50, 0.40, 0.60, 0.55, 0.70
), ncol = 2, byrow = TRUE) scenario5 < - matrix(c(0.15, 0.20, 0.20, 0.35, 0.25, 0.50, 0.20, 0.36, 0.35, 0.49, 0.40, 0.62, 0.45, 0.55, 0.55, 0.65, 0.75, 0.75 ), ncol = 2, byrow = TRUE) scenario6 < - matrix(c(0.50, 0.50, 0.55, 0.55, 0.65, 0.60, 0.55, 0.55, 0.70, 0.60, 0.75, 0.65, 0.65, 0.60, 0.80, 0.65, 0.85, 0.70 ), ncol = 2, byrow = TRUE) doseComb_to_mat(c(3,3), “matrix”) [, 1] [, 2] [, 3] [1,] 1 2 3 [2,] 4 5 6 [3,] 7 8 9

Based on the above scenarios, we constructed a total of 54 conditions, derived from 2 pre-specified toxicity and efficacy skeletons, 3 maximum sample sizes, 3 numbers of patients for determination of randomization phase, and 3 correlation parameters for efficacy and toxicity.

**Table:** 

orderings < - function(DLT1, DLT2, ORR1, ORR2){ input_Nphase < - c(10, 20, 30) input_corr < - c(0, −2.049, 0.814) input_N < - c(40, 50, 60) DLTs < - list(DLT1, DLT2) ORRs < - list(ORR1, ORR2)
conds < - list() i < - 1 for (s in 1:2){ for (n in 1:3){ for (np in 1:3){ for (c in 1:3){ conds[[i]] <- list(DLT=DLTs[[s]],ORR=ORRs[[s]], N = input_N[n], Nphase = input_Nphase[np], corr = input_corr[c]) i < - i + 1 } } } } return(conds)

Then, we specified two sets of toxicity and efficacy skeletons so that we could plug the skeletons into the orderings() function to store the 54 conditions into a list named conds.

**Table:** 

DLT_skeleton1 < - priorSkeletons(updelta = 0.045, target = 0.3, npos = 5, ndose = 9, model = “empiric”, prior=”normal”, beta_mean = 0) DLT_skeleton2 < - priorSkeletons(updelta = 0.06, target = 0.3, npos = 4, ndose = 9, model = “empiric”, prior=”normal”, beta_mean = 0) Efficacy_skeleton1 < - priorSkeletons(updelta = 0.045, target = 0.5, npos = 5, ndose = 9, model = “empiric”, prior=”normal”, beta_mean = 0) Efficacy_skeleton2 < - priorSkeletons(updelta = 0.06, target = 0.5, npos = 4, ndose = 9, model = “empiric”, prior=”normal”, beta_mean = 0) conds < - orderings(DLT1 = DLT_skeleton1, DLT2 = DLT_skeleton2, ORR1 = Efficacy_skeleton1, ORR2 = Efficacy_skeleton2) SC < - list(scenario1, scenario2, scenario3, scenario4, scenario5, scenario6)

Next, we built an output dataset with detailed names for 9 operating characteristics.

**Table:** 

output < - data.frame(Scenario = double(), Skeleton = double(), N = double(), nR = double(), corr = double(), safe = double(), target = double(), toxic = double(), avgSS = double(), prop_ODC = double(), stop_safety = double(), stop_futility = double(), o_DLT = double(), o_ORR = double()) colnames(output) <- c(“Scenario”, “Skeleton”, “N”, “nR”, “corr”, “Probability of recommending safe/ineffective combinations as ODC”, “Probability of recommending target combinations as ODC”, “Probability of recommending overly toxic combinations as ODC”, “Mean # of patients enrolled”, “Proportion of patients allocated to true ODC(s)”, “Proportion stopped for safety”, “Proportion stopped for futility”, “Observed DLT rate”, “Observed response rate”) conds = orderings(DLT1 = DLT_skeleton1, DLT2 = DLT_skeleton2, ORR1 = Efficacy_skeleton1, ORR2 = Efficacy_skeleton2)

The last step iterated through the 6 scenarios and 54 conditions, each with 1000 simulations. Results for each iteration were stored into an output dataset.

**Table:** 

for (s in 1:length(SC)){ for (c in 1:length(conds)){ curr <- SIM_phase_I_II(nsim=1000, Nmax=conds[[c]]$N, DoseComb = SC[[s]], input_doseComb_forMat = c(3,3), input_type_forMat = “matrix”, input_Nphase = conds[[c]]$Nphase, input_DLT_skeleton = conds[[c]]$DLT, input_efficacy_skeleton = conds[[c]]$ORR, input_DLT_thresh=0.3, input_efficacy_thresh=0.3,
input_cohortsize=1, input_corr=conds[[c]]$corr, input_early_stopping_safety_thresh = 0.33, input_early_stopping_futility_thresh = 0.2, input_model=“empiric”, input_para_prior=”normal”, input_beta_mean=0, input_beta_sd=sqrt(1.34), input_theta_mean=0, input_theta_sd=sqrt(1.34), random_seed = 42) currTmp < - data.frame(s, conds[[c]]$sklnum, conds[[c]]$N, conds[[c]]$Nphase, conds[[c]]$corr, curr$prob_safe, curr$prob_target, curr$prob_toxic, curr$mean_SS, curr$mean_ODC, curr$prob_stop_safety, curr$prob_stop_futility, curr$mean_DLT, curr$mean_ORR) output < - rbind(output, currTmp) } }

The output data were saved in the **crm12Comb**, named as dat, comprising 1296 rows (6 scenarios, 4 different combinations of link functions and prior distributions, 2 sets of skeletons, 3 maximum number of patients, 3 toxicity and efficacy correlations, and 3 subset number of patients) that represent each condition by 1000 simulations, along with 15 columns that contain 9 operating characteristics, other 6 columns including Scenario, Model for link function and prior distribution, N for maximum number of patients, Skeleton, Nphase for subset number of patients, and corr for toxicity and efficacy correlation to separate each condition. Various plots can be generated by the sample_plot() function to visually explore the relationships between operating characteristics and the maximum number of patients, number of patients for determination of the randomization phase, set of skeletons, and correlation between toxicity and efficacy binary outcomes by different scenarios and link functions.

**Fig 5:**
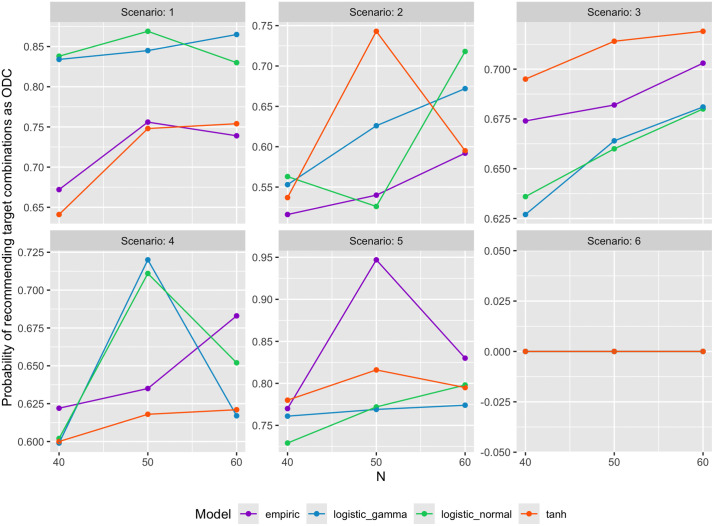
Probability of recommending target dose combinations as ODC among 1000 simulation trials.

**Fig 6:**
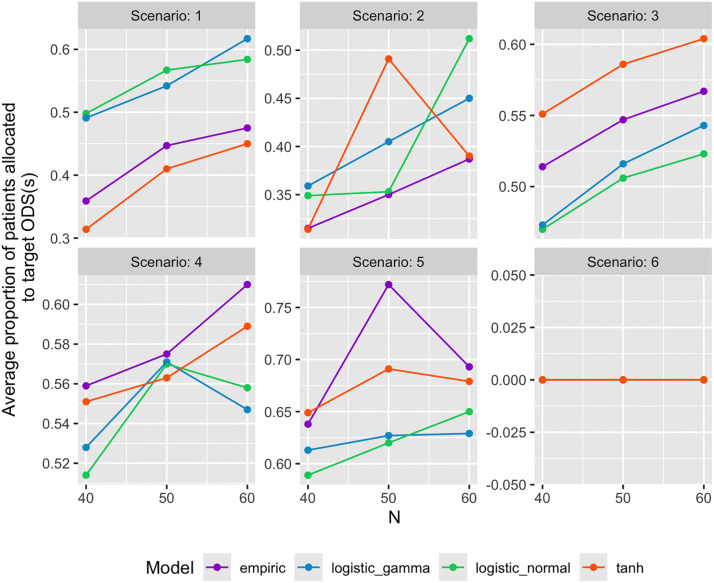
Average proportion of patients allocated to target ODS(s) among 1000 simulation trials.

Fig 5 and Fig 6 display sample plots derived from all simulations. These plots showed the probability of recommending target dose combinations as ODC and the average proportion of patients allocated to target ODC(s) among 1000 simulation trials versus the maximum number of patients, for all six scenarios, while fixing the other three variables. An observation from these plots is that no link function consistently outperforms the other three link functions, while tanh link function exhibited worse performance in Scenarios 2–5.

**Table:** 

sample_plot(examples_results, outcome = “prob_target”, outname = “Probability of ODC as target combinations”, N = NULL, nR = 20, Skeleton = 1, corr = 0)

**Table:** 

sample_plot(examples_results, outcome = “mean_ODC”, outname = “Average proportion of patients allocated to target ODS(s)”, N = NULL, nR = 20, Skeleton = 1, corr = 0)

## Discussion

In this paper, we introduced **crm12Comb**, an accessible R package tailored for conducting simulation studies on phase I/II adaptive design for drug combinations within a Bayesian CRM framework. **crm12Comb** offers the capability to perform not only a single trial simulation of the next patient allocation given current data, but also multiple trials to obtain a comprehensive preview of operating characteristics even before a trial starts. We also demonstrated how to execute multiple simulation studies with a range of input parameters.

Building upon the algorithm using the one-parameter empiric link function with standard normal prior distribution, as suggested in [23], **crm12Comb** incorporates three commonly used link functions in clinical research, enhancing the package’s versatility. Users can choose from various link functions with self-determined values of parameters for different prior distributions, including the one-parameter empiric model with normal or gamma priors, one-parameter hyperbolic tangent model with exponential priors, one-parameter logistic model with normal or gamma priors, and two-parameter logistic model with normal or gamma priors. This selection allows for the customization of prior distributions based on historical data, thereby improving the package’s utility for diverse clinical scenarios.

In addition to versatile link functions with priors, **crm12Comb** enables users to tailor various aspects of their phase I/II drug combination trial based on CRM design, including the maximum sample size, number of dose combinations, pre-defined true toxicity and efficacy probabilities for dose combinations, cohort size, the number of patients for determination of randomization phase, pre-specified toxicity and efficacy skeletons, correlation between toxicity and efficacy binary outcomes, DLT and efficacy thresholds, and thresholds for early stopping.

Furthermore, **crm12Comb** facilitates the creation of visualization, including plots for single-trial patient sequential enrolment with toxicity/efficacy outcomes, single-trial patient allocation by dose combinations, and the overall dose combination identified as ODC(s) among all simulations. Sample data and plots comparing different link functions are also provided within the package to serve as practical references.

A current limitation of the package is the lack of available data from real-world phase I/II drug combination trials, particularly patient-level toxicity, efficacy, and allocation information. Our review identified only two trials aligning with our package’s focus; however, neither provided the detailed patient-level outcomes or trial design specifics needed to reproduce the trial process. To address this gap, our package crm12Comb provides flexible simulation inputs that allow users to mimic realistic trial settings and explore practical scenarios. As relevant patient-level data become available, future development will focus on integrating real trial examples, to further demonstrate and evaluate the package’s practical utility.

## Supporting information

S1 File Detailed toxicity and efficacy estimation of two-parameter logistic link function. (DOCX)
