# Supplementary material for: crm12comb: Phase I/II adaptive design for drug combinations based on CRM design through R
Source: PLoS One. 2025 Nov 10;20(11):e0336146. doi: 10.1371/journal.pone.0336146 (PMC12599976; doi:10.1371/journal.pone.0336146)
Supplement: S1 File — (DOCX) [file pone.0336146.s001.docx]

### **Toxicity estimation of** **two-parameter logistic link function**

For the two-parameter logistic link function with normal prior distributions, the toxicity estimation is updated at first with link function

$$F_{m}\left( d_{i},\alpha_{T},\beta\right)=\frac{1}{1+\exp\left( -\alpha_{T}-\exp\left( \beta\right)d_{im} \right)}$$

with two parameters $\alpha_{T}$ and $\beta$. $d_{im}$ is the scaled transformation from the toxicity skeleton by

$d_{im}=\left[ \log\left( \frac{p_{im}}{1-p_{im}} \right)-\exp\left( \bar{\alpha}_{T} \right) \right]/{\exp\left( \bar{\beta} \right)}$,

where $\bar{\alpha}_{T}$ and $\bar{\beta}$ are expected values from normal prior distributions $g_{1}\left( \alpha_{T} \right)$ and $g_{2}\left( \beta\right)$. Then, the likelihood function in Equation (3.1) will be updated to

$L_{m}\left( \alpha_{T},\beta| \mathcal{T}_{\mathcal{j}} \right)=\prod_{l=1}^{j} \{F_{m}\left( x_{l},\alpha_{T},\beta\right){\}}^{y_{l}}\{1-F_{m}\left( x_{l},\alpha_{T},\beta\right){\}}^{1-y_{l}}$.

The posterior joint density function for $\alpha_{T}$ and $\beta$ based on Equation (3.2) is updated to

$P\left( \alpha_{T},\beta| \mathcal{T}_{\mathcal{j}} \right)=\frac{L_{m}\left( \alpha_{T},\beta| \mathcal{T}_{\mathcal{j}} \right)g_{1}\left( \alpha_{T} \right)g_{2}\left( \beta\right)}{\int_{\mathcal{A}_{\mathcal{T}}} \int_{\mathcal{B}} L_{m}\left( \alpha_{T},\beta| \mathcal{T}_{\mathcal{j}} \right)g_{1}\left( \alpha_{T} \right)g_{2}\left( \beta\right)d\beta d\alpha_{T}}$,

where the marginal posterior density functions are

$P\left( \alpha_{T} | \mathcal{T}_{\mathcal{j}} \right)=\int_{\mathcal{B}} P\left( \alpha_{T},\beta| \mathcal{T}_{\mathcal{j}} \right)d\beta, P\left( \beta| \mathcal{T}_{\mathcal{j}} \right)=\int_{\mathcal{A}_{\mathcal{T}}} P\left( \alpha_{T},\beta| \mathcal{T}_{\mathcal{j}} \right)d\alpha_{T}.$ (5)

Based on Equation (3.5), the estimated $\hat{\alpha}_{T_{jm}}$ and $\hat{\beta}_{jm}$ can be further generated by

$\hat{\alpha}_{T_{jm}} =\int_{\mathcal{A}_{\mathcal{T}}} \alpha_{T}P\left( \alpha_{T} | \mathcal{T}_{\mathcal{j}} \right)d\alpha_{T},\quad\hat{\beta}_{jm}=\int_{\mathcal{B}} \beta P\left( \beta| \mathcal{T}_{\mathcal{j}} \right)d$.

The posterior density for $m$ based on Equation (3.3) is updated to

$w\left( m | \mathcal{T}_{\mathcal{j}} \right)=\frac{\tau\left( m \right)\int_{\mathcal{A}_{\mathcal{T}}} \int_{\mathcal{B}} L_{m}\left( \alpha_{T},\beta| \mathcal{T}_{\mathcal{j}} \right)g_{1}\left( \alpha_{T} \right)g_{2}\left( \beta\right)d\beta d\alpha_{T}}{\sum_{m=1}^{M} \tau\left( m \right)\int_{\mathcal{A}_{\mathcal{T}}} \int_{\mathcal{B}} L_{m}\left( \alpha_{T},\beta| \mathcal{T}_{\mathcal{j}} \right)g_{1}\left( \alpha_{T} \right)g_{2}\left( \beta\right)d\beta d\alpha_{T}}$. (6)

From Equation (3.6), a single drug combination $m^{*}$ is chosen with the largest posterior probability $m^{*}=\arg\max_{m} w\left( m | \mathcal{T}_{\mathcal{j}} \right), m=1,\ldots,M,$ which can be used to define the acceptable set for the following efficacy estimation.

### **Efficacy estimation of two-parameter logistic link function**

For the two-parameter logistic link function with normal prior distributions, the efficacy estimation is updated at first by link function $F_{k}\left( d_{i},\alpha_{E},\theta\right)=\frac{1}{1+\exp\left( -\alpha_{E}-\exp\left( \theta\right)d_{ik} \right)}$ with two parameters $\alpha_{E}$ and $\theta$. $d_{ik}$ is the scaled transformation from the toxicity skeleton by $d_{ik}=\left[ \log\left( \frac{q_{ik}}{1-q_{ik}} \right)-\exp\left( \bar{\alpha}_{E} \right) \right]/{\exp\left( \bar{\theta} \right)}$, where $\bar{\alpha}_{E}$ and $\bar{\theta}$ are expected values from normal prior distributions $h_{1}\left( \alpha_{E} \right)$ and $h_{2}\left( \theta\right)$. Then, the likelihood function will be updated to

$L_{k}\left( \alpha_{E},\theta| \mathcal{E}_{\mathcal{j}} \right)=\prod_{l=1}^{j} \{F_{k}\left( x_{l},\alpha_{E},\theta\right){\}}^{Z_{l}}\{1-F_{k}\left( x_{l},\alpha_{E},\theta\right){\}}^{1-Z_{l}}$.

The posterior joint density function for $\alpha_{E}$ and $\theta$ is updated to

$P\left( \alpha_{E},\theta| \mathcal{E}_{\mathcal{j}} \right)=\frac{L_{k}\left( \alpha_{E},\theta| \mathcal{E}_{\mathcal{j}} \right)h_{1}\left( \alpha_{E} \right)h_{2}\left( \theta\right)}{\int_{\mathcal{A}_{\mathcal{E}}} \int_{\Theta} L_{k}\left( \alpha_{E},\theta| \mathcal{E}_{\mathcal{j}} \right)h_{1}\left( \alpha_{E} \right)h_{2}\left( \theta\right)d\theta d\alpha_{E}}$,

where the marginal posterior density functions are

$$P\left( \alpha_{E} | \mathcal{E}_{\mathcal{j}} \right)=\int_{\Theta} P\left( \alpha_{E},\theta| \mathcal{E}_{\mathcal{j}} \right)d\theta, P\left( \theta| \mathcal{E}_{\mathcal{j}} \right)=\int_{\mathcal{A}_{\mathcal{E}}} P\left( \alpha_{E},\theta| \mathcal{E}_{\mathcal{j}} \right)d\alpha_{E}.$$

Then, the estimated $\hat{\alpha}_{E_{jk}}$ and $\hat{\theta}_{jk}$ can be further generated by

$\hat{\alpha}_{E_{jk}} =\int_{\mathcal{A}_{\mathcal{E}}} \alpha_{E}P\left( \alpha_{E} | \mathcal{E}_{\mathcal{j}} \right)d\alpha_{E},\quad\hat{\theta}_{jk}=\int_{\Theta} \theta P\left( \theta| \mathcal{E}_{\mathcal{j}} \right)d\theta$.

The posterior density for $k$ is updated to

$w\left( k | \mathcal{E}_{\mathcal{j}} \right)=\frac{\xi\left( k \right)\int_{\mathcal{A}_{\mathcal{E}}} \int_{\Theta} L_{k}\left( \alpha_{E},\theta| \mathcal{E}_{\mathcal{j}} \right)h_{1}\left( \alpha_{E} \right)h_{2}\left( \theta\right)d\theta d\alpha_{E}}{\sum_{k=1}^{K} \xi\left( k \right)\int_{\mathcal{A}_{\mathcal{E}}} \int_{\Theta} L_{k}\left( \alpha_{E},\theta| \mathcal{E}_{\mathcal{j}} \right)h_{1}\left( \alpha_{E} \right)h_{2}\left( \theta\right)d\theta d\alpha_{E}}$.

Subsequently, the estimate for $\alpha_{E}$ and $\theta$, $\hat{\alpha}_{E_{jk}}$ and $\hat{\theta}_{jk}$, can be obtained. For next enrolled patient or cohort of patients, the order $k^{*}$ with the highest posterior probability is selected, and the efficacy probability for each drug combination under ordering $k^{*}$ is estimated as:

$\hat{\pi_{E}}\left( d_{i} \right)=F_{k^{*}}\left( d_{i},{\hat{\alpha}_{E_{jk^{*}}}, \hat{\theta}}_{jk^{*}} \right)$,

Then the $\hat{\pi_{E}}\left( d_{i} \right)$ can be used to guide the allocation of new patient or patient cohort.
